# Supplementary material for: Engineering Cortical Networks: An Open Platform for Controlled Human Circuit Formation and Synaptic Analysis In Vitro
Source: Adv Healthc Mater. 2025 Oct 24;14(31):e00857. doi: 10.1002/adhm.202500857 (PMC12683197; doi:10.1002/adhm.202500857)
Supplement: Supplementary file 1 — Supporting Information [file ADHM-14-0-s001.docx]

**Supplementary Information**

**Engineering Cortical Networks: An Open Platform for Controlled Human Circuit Formation and Synaptic Analysis In Vitro**

*Pacharaporn Suklai^1,2,3^, Taylor Minckley^1,2,3^*, *Cathleen Hagemann^1,2,3^, Karolina Faber^4^, Rosalind Norkett^3^, Ludovica Guetta^1,2,3^, Kelly O’Toole^1,2,3^, Bethany Geary^4^, Michael J. Devine^3,5^, Andrea Serio^1,2,3*^*

**Affiliation**

**1.**  Department of Basic and Clinical Neuroscience, Institute of Psychiatry Psychology & Neuroscience, King’s College London, London, UK.

**2.** UK Dementia Research Institute at King’s College London, London, UK.

**3.** The Francis Crick Institute, London, UK

**4.** Medical Research Council (MRC) Protein Phosphorylation and Ubiquitylation Unit, School of Life Sciences, University of Dundee, Dundee, UK

**5.** Department of Clinical and Movement Neurosciences, UCL Queen Square Institute of Neurology, University College London, London WC1N 3BG, UK

* = corresponding author ([andrea.serio@kcl.ac.uk](mailto:andrea.serio@kcl.ac.uk) / [andrea.serio@crick.ac.uk](mailto:andrea.serio@crick.ac.uk))


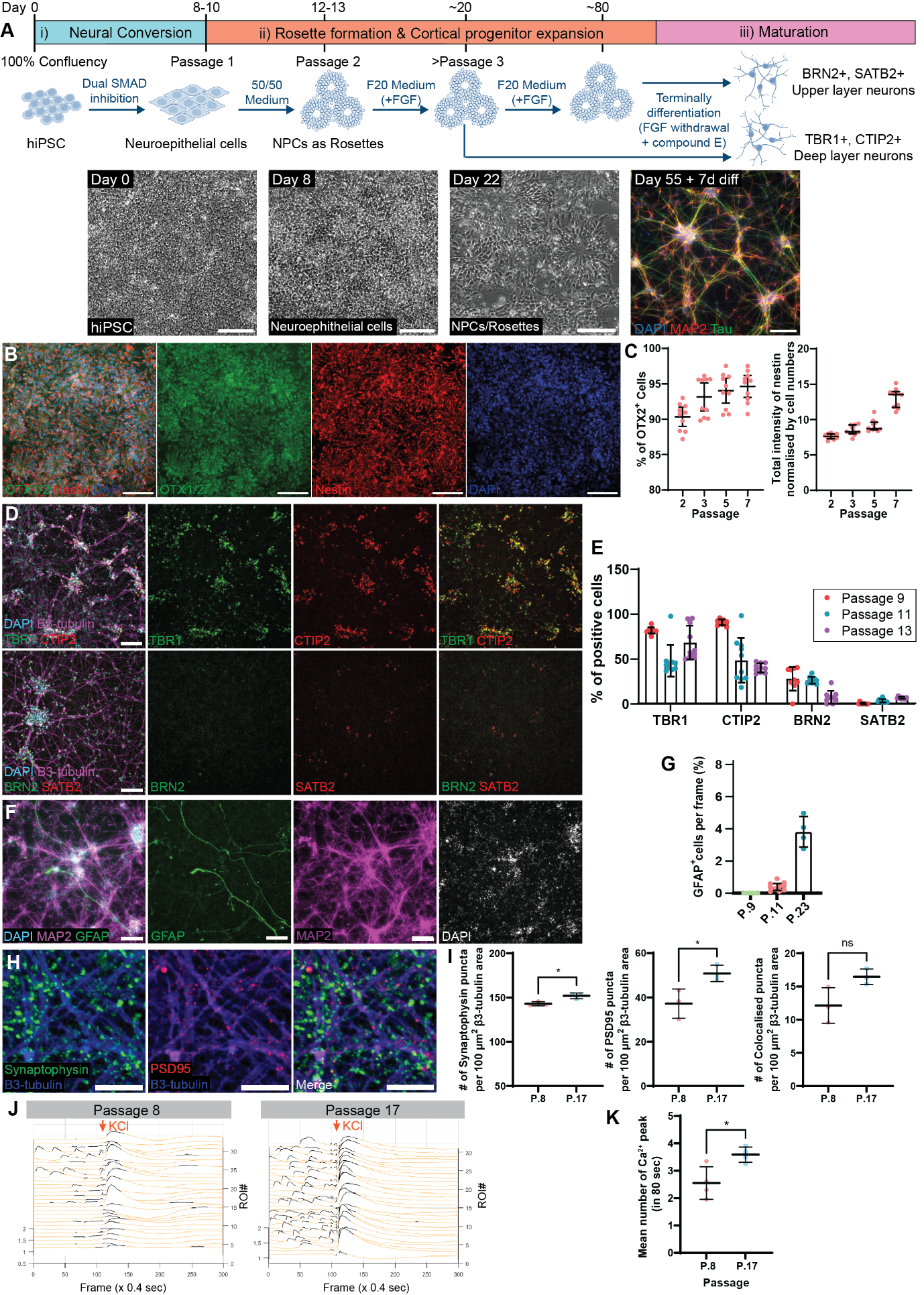


**Supplementary Figure 1**

**Generation and characterization of iPSC-derived cortical neurons A**) Schematic overview of cortical neuron induction and differentiation from iPSCs, containing 3 main phases: i) neural conversion, ii) neural rosette formation and proliferation of cortical neuron progenitors, and iii) terminal differentiation and maturation. Representative images show the progression from iPSCs (Day 0) to neuroepithelial cells (Day 8), followed by the formation of neural progenitor cells organized into 'rosette' structures (Day 22). Upon FGF withdrawal, these progenitors terminally differentiate into cortical neurons (Day 55 + 7 days). Staining includes DAPI for nuclei, MAP2 for dendrites, and Tau for axons. Scale bar: 100 μm. **B**) Representative images of cortical neuron progenitors expressing OTX1/2, a forebrain cortical progenitor marker, and Nestin, a general neural marker. Scale bar: 100 μm. **C**) Quantification of OTX1/2 positive cells (left) and Nestin expression, presented as total Nestin intensity normalized to the number of cells (right) in cortical neuron progenitor cultures across different passages. Data points represent each field of view (FOV) from 2 technical replicates, with results shown as the median ± interquartile range. **D**) Representative images of cortical neurons at passage 13, terminally differentiated for 7 days, stained for cortical neuron subtype transcription factors: TBR1 and CTIP2 (deeper layer) and BRN2 and SATB2 (upper layer). Scale bar: 100 μm. **E**) Percentage of cells positive for each transcription marker across different passages. Data points represent each FOV from 2 technical replicates, with results shown as mean ± S.D. **F**) Representative images of cortical neuron culture at passage 11, terminally differentiated for 7 days, stained for astrocyte marker GFAP, neuronal markers MAP2, and DAPI for nuclei. Most DAPI-positive cells are MAP2-positive neurons. Bright, small, rounded DAPI-only nuclei likely represent dead cells; other non-neuronal cell types are not expected in the culture. Scale bar: 100 μm. **G**) Percentage of GFAP positive cells in culture across different passages. Data points represent each FOV from 2 technical replicates, with results shown as mean ± S.D. **H**) Representative images of cortical neurons from passage 17 (day 70), showing the expression of synaptic proteins synaptophysin and PSD95, with β3-tubulin as a neuronal cytoskeleton marker. Scale bar: 10 µm. **I**) Quantification of synaptic puncta: pre-synaptic puncta (synaptophysin, left), post-synaptic puncta (PSD95, middle), and colocalised puncta (right) per 100 μm^2^ β3-tubulin. Data represent well averages from 1 independent experiment with 3 technical replicates. Cell line: Ctrl3. Synaptophysin data are expressed as median ± interquartile range and analysed using the Mann- Whitney test, while PSD95 and colocalized puncta are expressed as mean ± SD, and analysed using an unpaired t-test (* P<0.05, ns: non-significant). **J**) Representative calcium spike traces from individual regions of interest (ROIs) drawn around the soma of cortical neurons, terminally differentiated for 14 days from progenitors at passage 8 (day 38) and passage 17 (day 70). Neurons were stimulated with 30 mM KCl at an indicated arrow. **K**) Quantification of the total number of calcium spikes at baseline over an 80-second period. Data represent well averages from 1 independent experiment with 4 technical replicates. Cell line: Ctrl3. Results are expressed as mean ± SD, using unpaired t test to test for significance (* P<0.05, ns: non-significant).


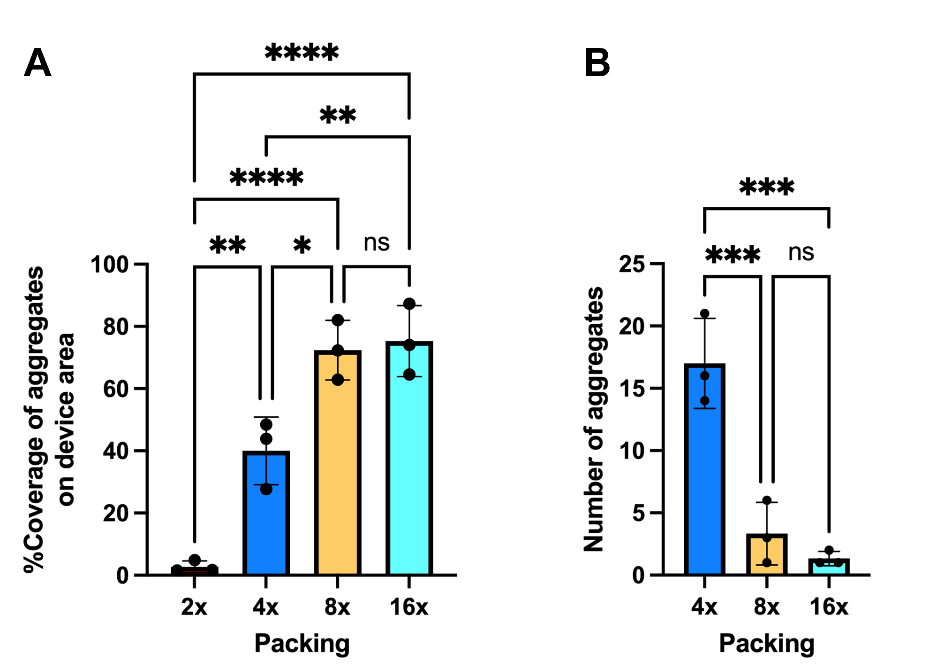


**Supplementary Figure 2**

**Quantification of neuronal aggregate characteristics across packing densities.** A) Percentage of aggregate coverage across 2x, 4x, 8x, and 16x neuronal packing densities. Higher packing densities resulted in increased area coverage by neuronal aggregates. B) Number of aggregates formed per node at 4x, 8x, and 16x packing. Aggregate number decreased with increasing density, reflecting a shift from dispersed small clusters to fewer and larger aggregates. In both graphs, data points represent individual replicates, expressed as mean ± SD. Statistical analysis was performed using one-way ANOVA test with Tukey’s multiple comparisons test. ****P<0.0001, ***P<0.001, **P <0.01, *P <0.05, ns: non-significant. Data were obtained from 1 independent experiment with 3 technical replicates. Cell line: Ctrl3.


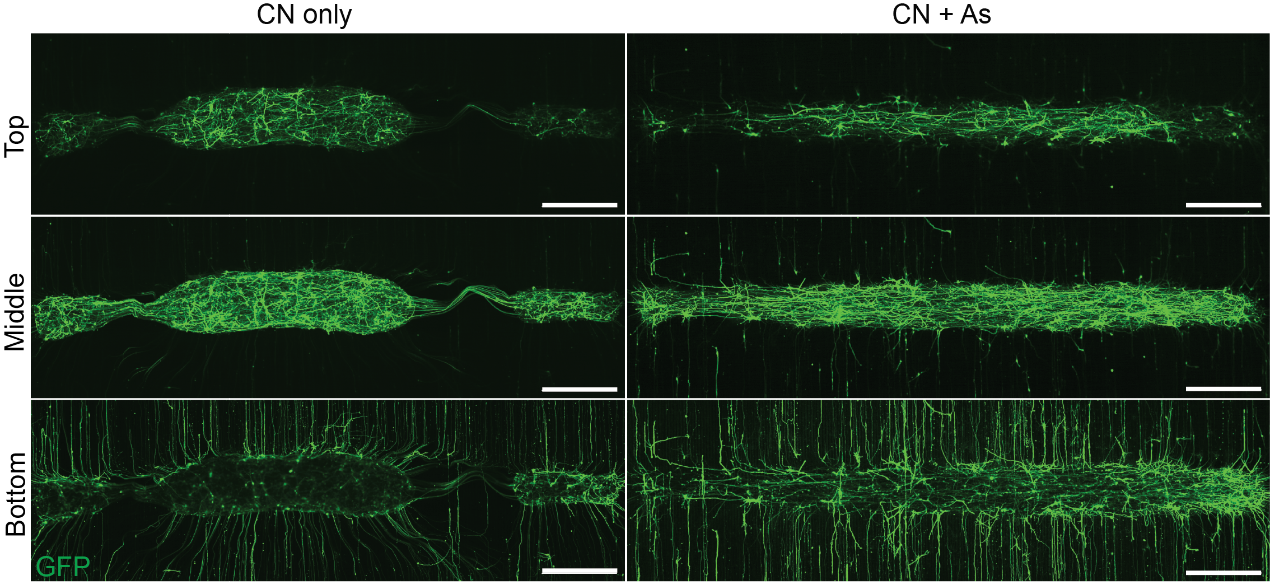


**Supplementary Figure 3**

**Internal organization of cortical neuron neurites.** Representative images of neuronal nodes with sparse GFP cortical neurons in conditions with and without astrocytes at the top, middle, and bottom focal planes. GFP shows the alignment of neurites inside the nodes and on the topography. Scale bar: 500 μm.


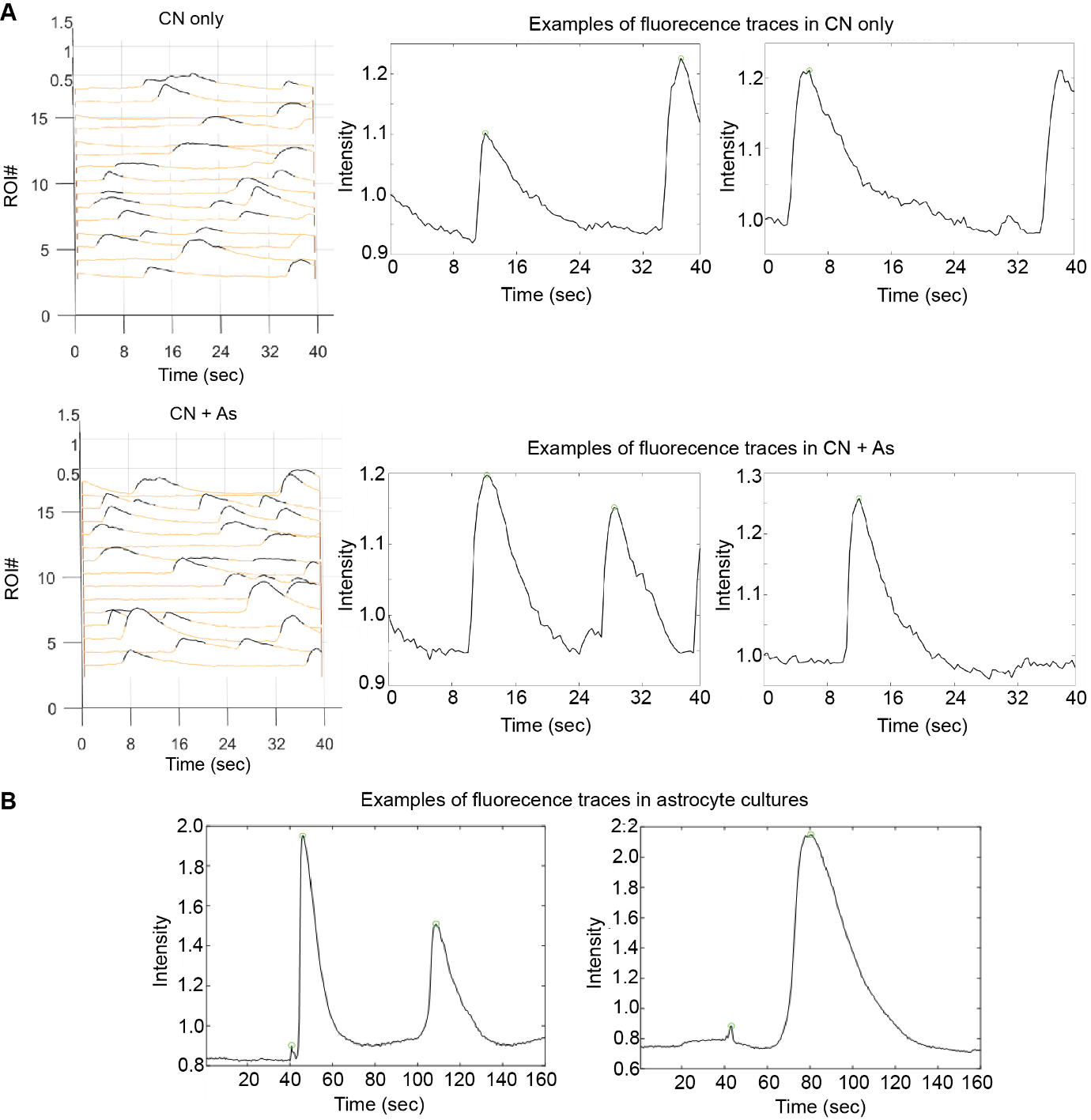


**Supplementary Figure 4**

**Representative calcium traces** A) Traces from ROIs drawn around the somas of cortical neurons terminally differentiated for 14 days with examples of calcium trace in individual ROIs from cortical neuron-only nodes and cortical neurons co-cultured with astrocytes nodes. Neuronal calcium activity typically shows rapid spiking, while astrocytes display slower, broader calcium waves. B) Representative calcium trace from an astrocyte-only culture following ATP stimulation at 40 seconds into the recordings.

**
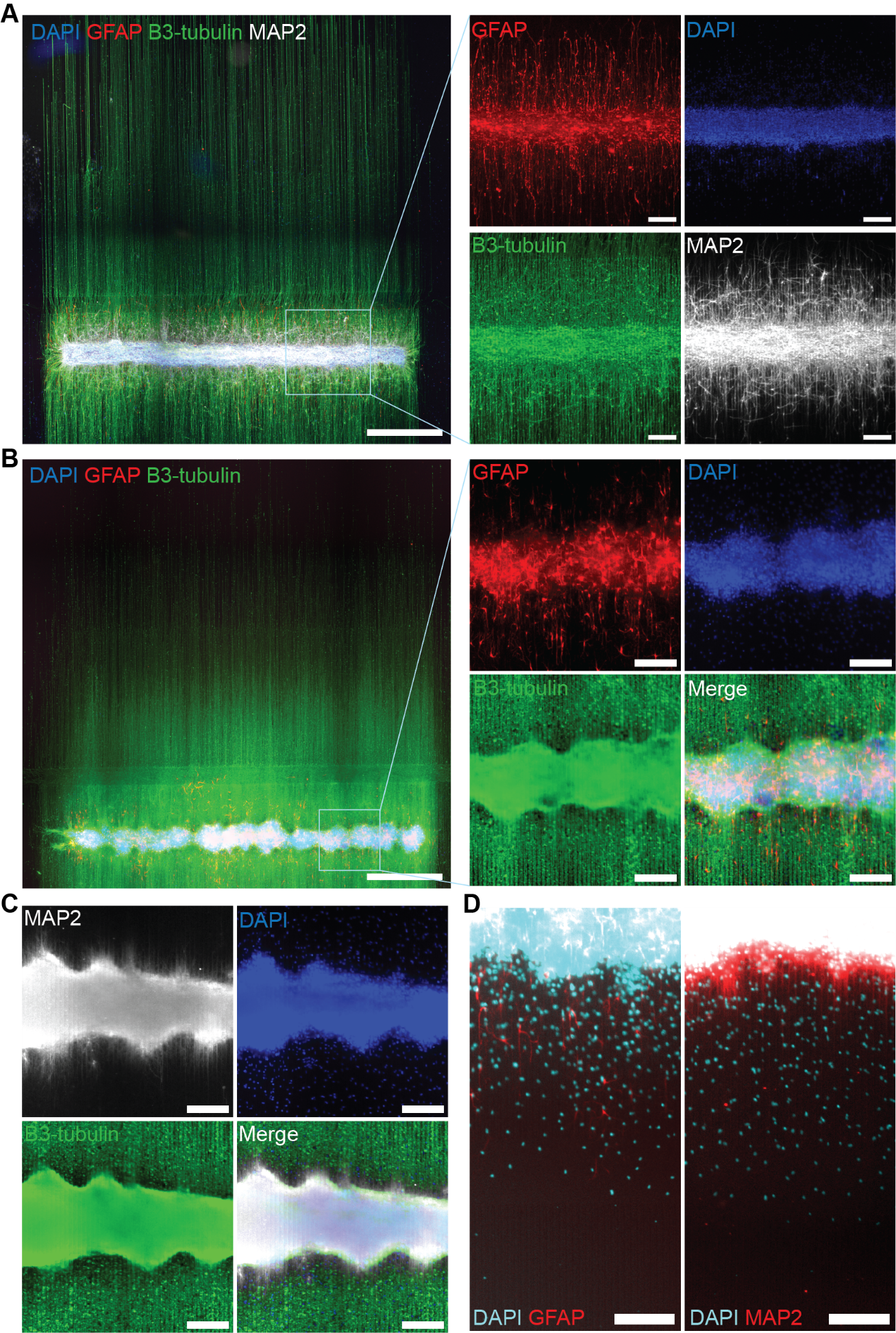
**

**Supplementary Figure 5**

**Validation of neuronal node construction in mouse primary neuron culture.** A) Neuronal node derived from human iPSC cortical neurons and astrocytes plated at 16x hexagonal packing. Scale bar: 1000 μm. The dashed square shows a zoom-in view showing the organization of astrocytes (GFAP), nuclei (DAPI), axons (β3-tubulin) and dendrites (MAP2) within the node, scale bar: 200 μm. B-D) Neuronal nodes from primary mouse neurons E16.5 plated at different hexagonal packing: B) Node plated using cell numbers calculated for 8x hexagonal packing, scale bar: 1000 μm. The dashed square shows a zoom-in view showing the organization of astrocytes (GFAP), nuclei (DAPI), and axons (β3-tubulin), scale bar: 200 μm. C) Node plated using cell numbers calculated for 16x hexagonal packing, stained for dendrites (MAP2), nuclei (DAPI) and axons (β3-tubulin). Scale bar: 200 μm. D) Mouse neuronal nodes showing MAP2-positive neurons predominantly contained within the node, with some astrocytes and unidentified cells migrating around the periphery. Scale bar: 200 μm.


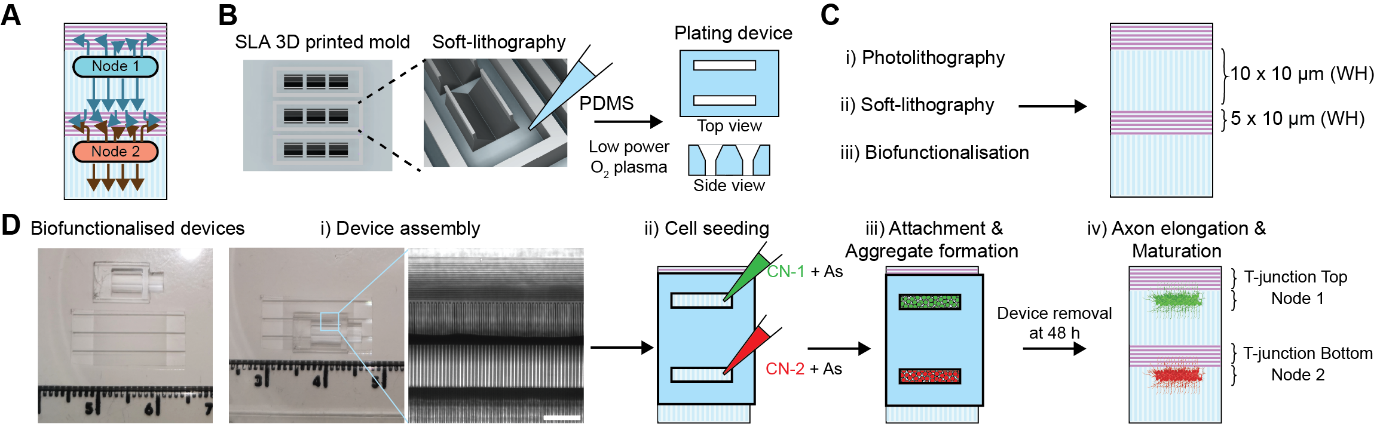


**Supplementary Figure 6**

**Assembly of cortical circuit on BIOCONNET platform** A) Schematics of the platform showing topographical cues that control neurite directionality to bias overlapped neurites from two nodes at T-junction. B) PDMS stencil of 2-pocket plating device is generated from 3D-printed mould and exposed to O_2_ plasma before cell plating. C) PDMS stencil of T-shape micropattern is generated from photolithography-based mould and biofunctionalized with O_2_ plasma, coated with PDL and laminin before cell plating. D) The plating device is placed on the micropattern, in which the pocket aligned approximately 200-250 µm below horizontal grooves (i). Scale bar: 200 µm. For cell seeding, two cortical neuron progenitor populations, mixed with astrocyte progenitors are seeded into each pocket and allowing aggregate formation for 48 hours before the device removal. Cells undergo neurite extension and network formation under differentiation and maturation processes.


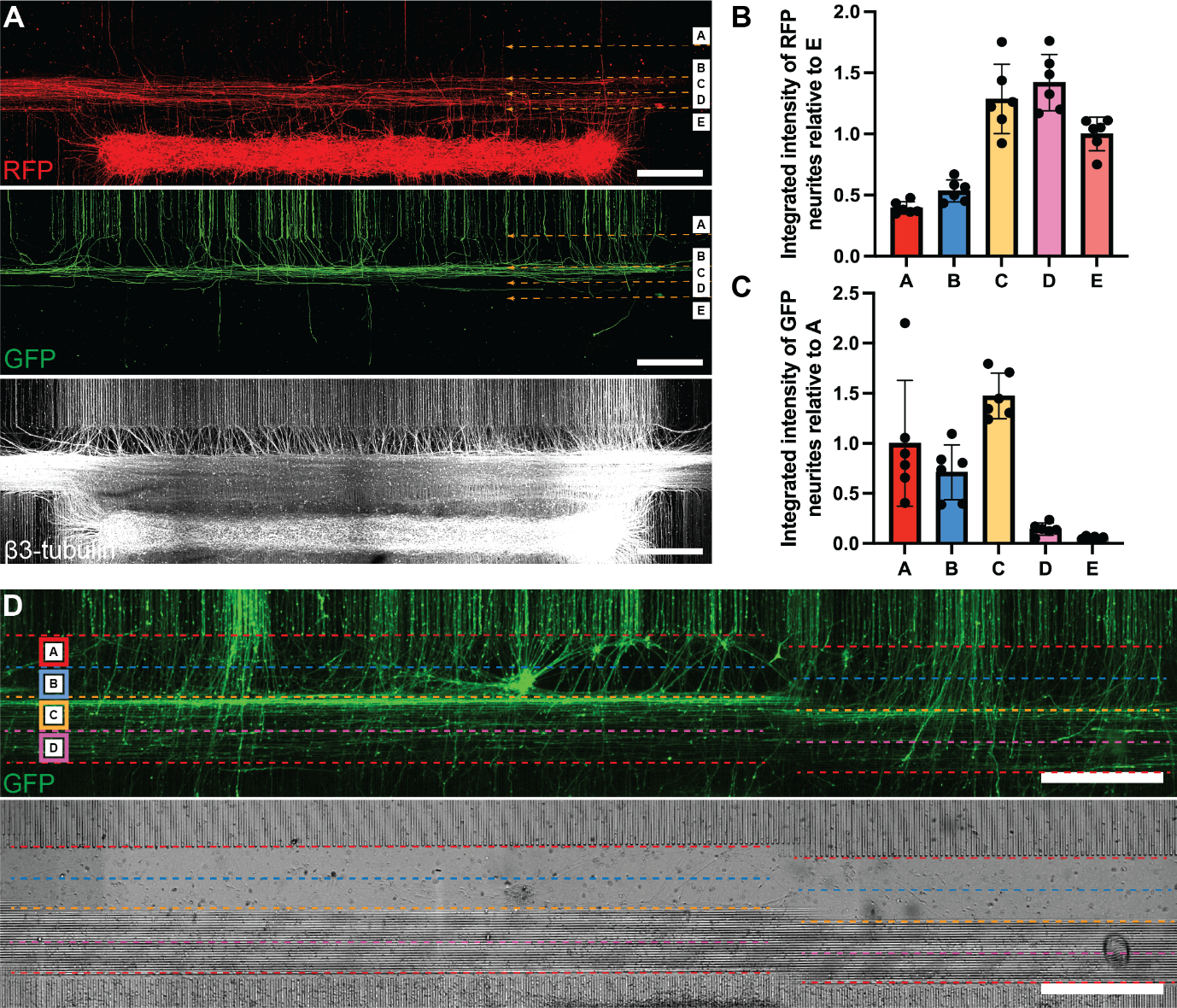


**Supplementary Figure 7**

**Neurites at T-junction area** A) Representative image of T-junction between two nodes, showing RFP-labeled neurites projecting from the bottom nodes and GFP-labeled axons from the top node extending toward the T-junction. Scale bar: 500 µm. B) Quantification of RFP neurite occupancy in the T-junction region, based on the integrated intensity of RFP signal within the ROIs indicated in (A), normalized to the intensity in section E, which represents the total neurite projection from the bottom node. Data are shown as mean ± SD for each ROI. C) Quantification of GFP neurite occupancy in the T-junction region, based on the integrated intensity of GFP signal within the ROIs indicated in (A), normalized to the intensity in section A, which represents the total GFP neurite input into the T-junction. Data are shown as mean ± SD for each ROI. D) Overview image of the T-junction area at 14 days of differentiation, showing the alignment of GFP-labeled axons from the top node approaching neurites from the bottom node. the boundary separating regions where ROIs were selected for directionality analysis. Scale bar: 500 µm.


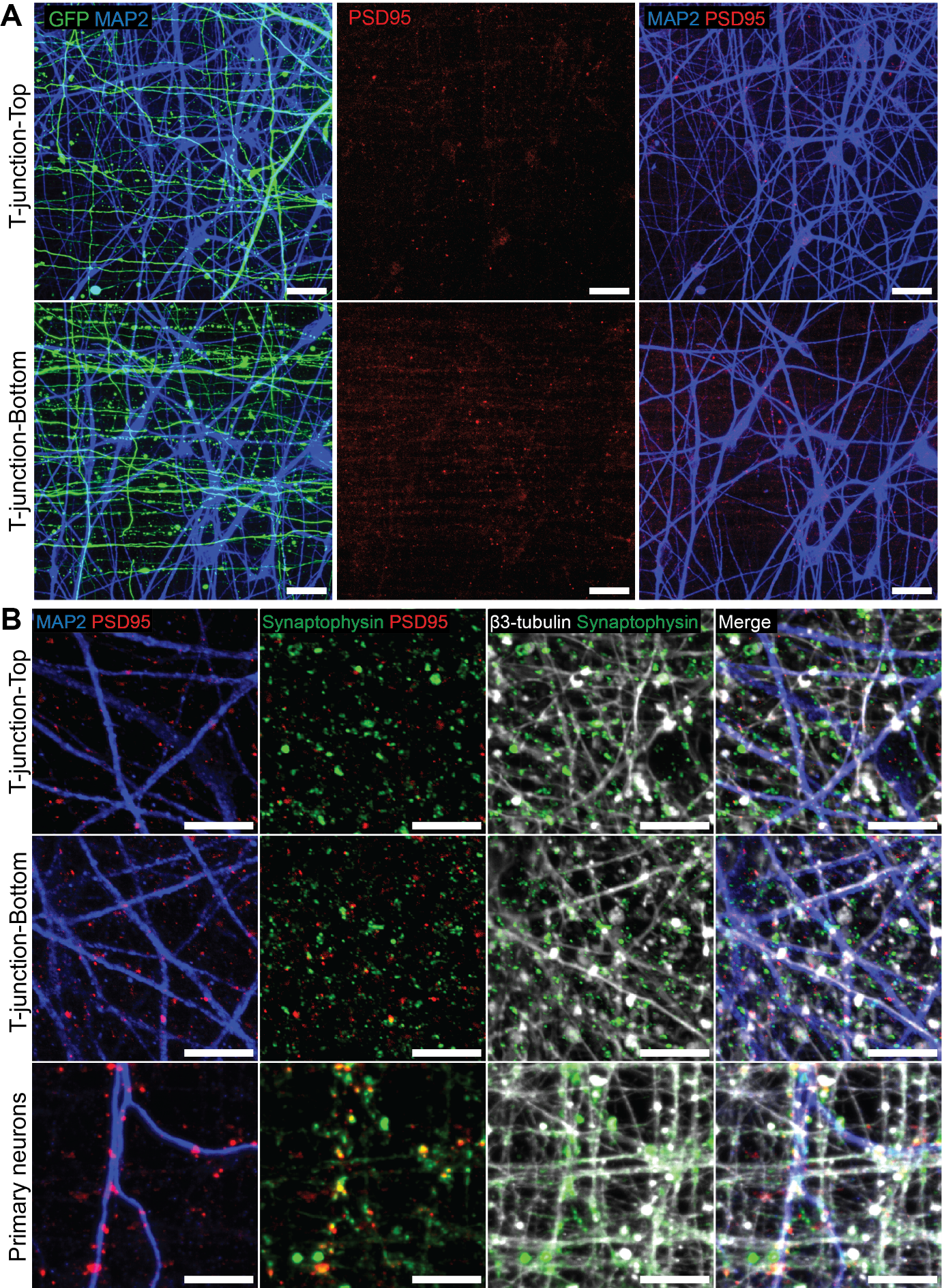


**Supplementary Figure 8**

**Synaptic protein staining** A) Representative images of the T-junction-top and T-junction-bottom areas in the circuits GFP-labeled neurons from the top node projecting toward non-fluorescent neurons in the bottom node. Scale bar: 20 μm. B) Representative synaptic staining in human iPSC-derived cortical neurons at the T-junction. As a positive control, staining was also performed on primary mouse cortical neurons derived from E16.5 embryos, which reliably reach synaptic maturity. This demonstrates that the staining protocol and antibody conditions are optimized to detect synaptic puncta under mature neuronal conditions. Scale bar: 10 μm.


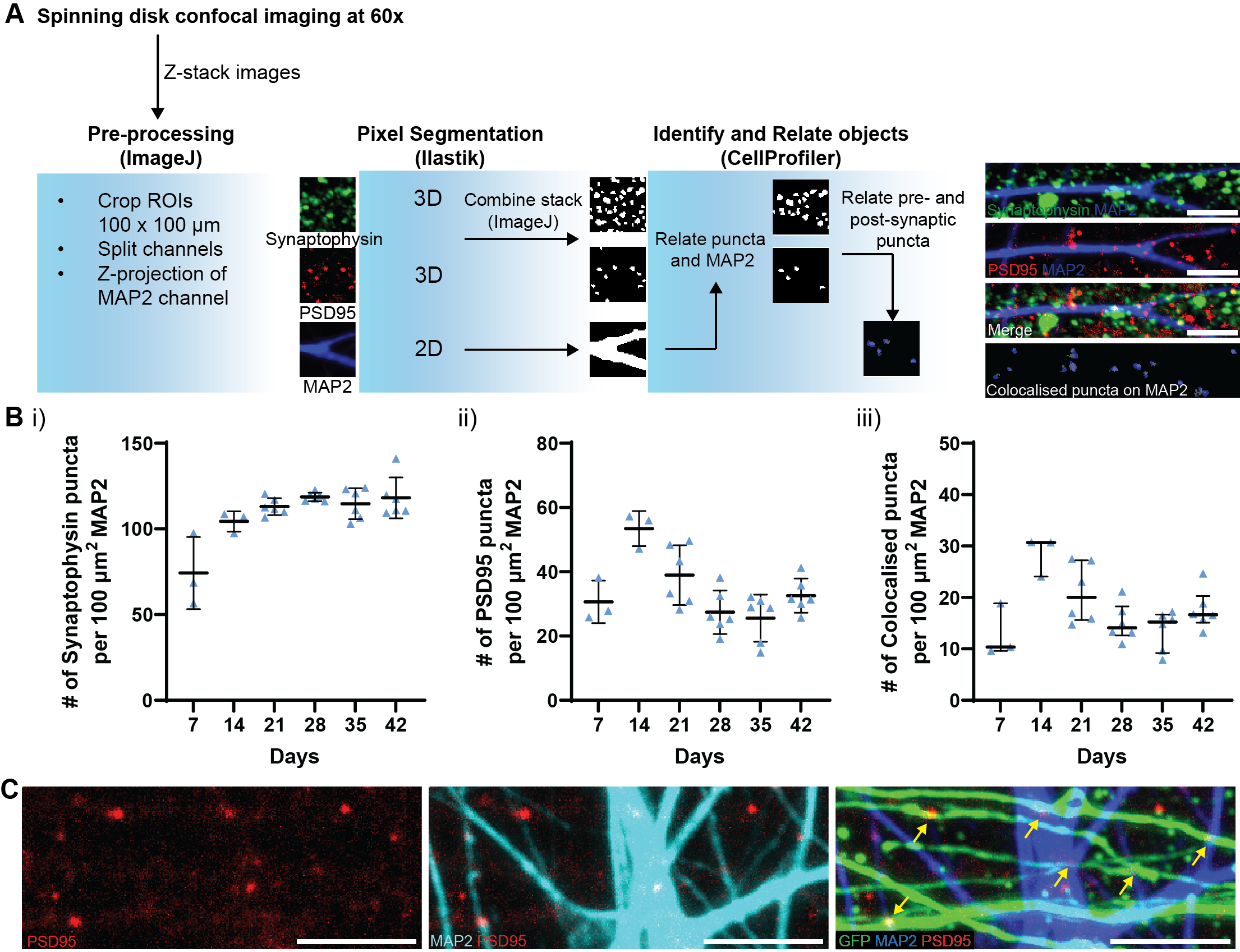


**Supplementary Figure 9**

**Analysis of synaptic puncta density and connectivity** A) Image analysis pipeline for synapse quantification. Z-stack images of pre-and post-synaptic protein: synaptophysin, PSD95, and neuronal structure: MAP2 were acquired from confocal microscopy with 60x magnification. Pre-processing of images was performed before object segmentation based on image pixel intensity. Each puncta object was first identified on MAP2 mask and subjected to overlapping object identification between pre-and postsynaptic puncta. Scale bar: 5 μm. B) Cortical neurons were culture on 2D ibidi glass chamber and analysed for the number of (i) pre-synaptic puncta: synaptophysin, (ii) post-synaptic puncta: PSD95, and (iii) colocalised puncta per 100 μm^2^ MAP2 positive dendrite areas, during the differentiation period from 7 to 42 days. Data points represent well averages from 2 independent experiments with 3 technical well replicates, cell line: Ctrl3, expressed as median ± interquartile range. C) Representative images of colocalization (yellow arrow) between GFP axons from the top node and PSD95 puncta on dendrites (MAP2) of the bottom node. Scale bar: 10 μm.


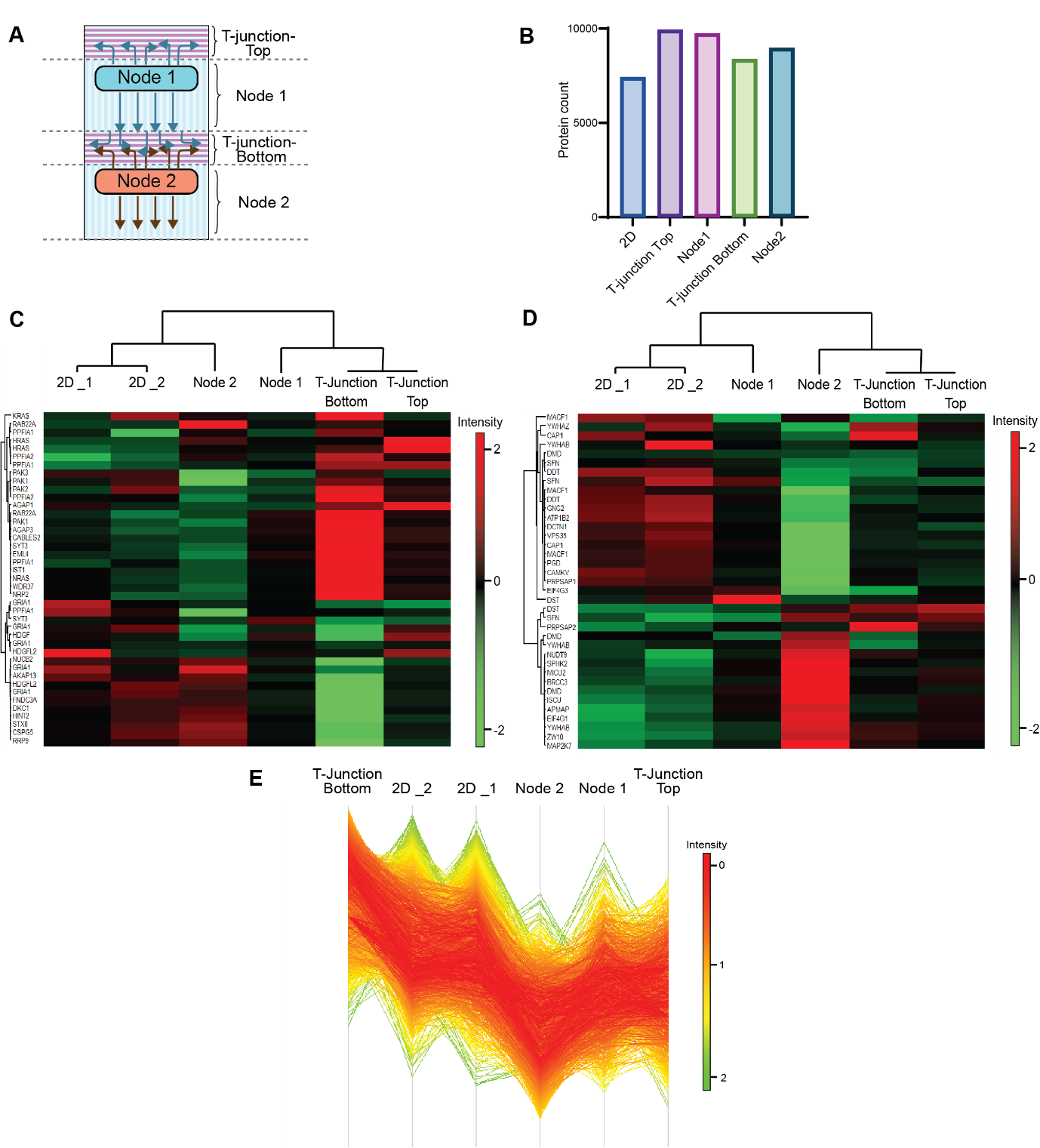


**Supplementary Figure 10**

**Proteomics analysis of the circuits** A) Schematic circuits on the platform, with dashed lines indicating where each fraction was cut for proteomic analysis. B) Counts of proteins analysed using Astral Orbitrap system from isolated regions of cortical networks. C) Heatmap of main contributing proteins to component 1, with samples and proteins clustered by Spearman correlation. D) Heatmap of main contributing proteins to component 2, with samples and proteins clustered by Spearman correlation. E) Density plot of the selected cluster (see **Figure 6H**), identifying upregulated proteins in T-junction Bottom.


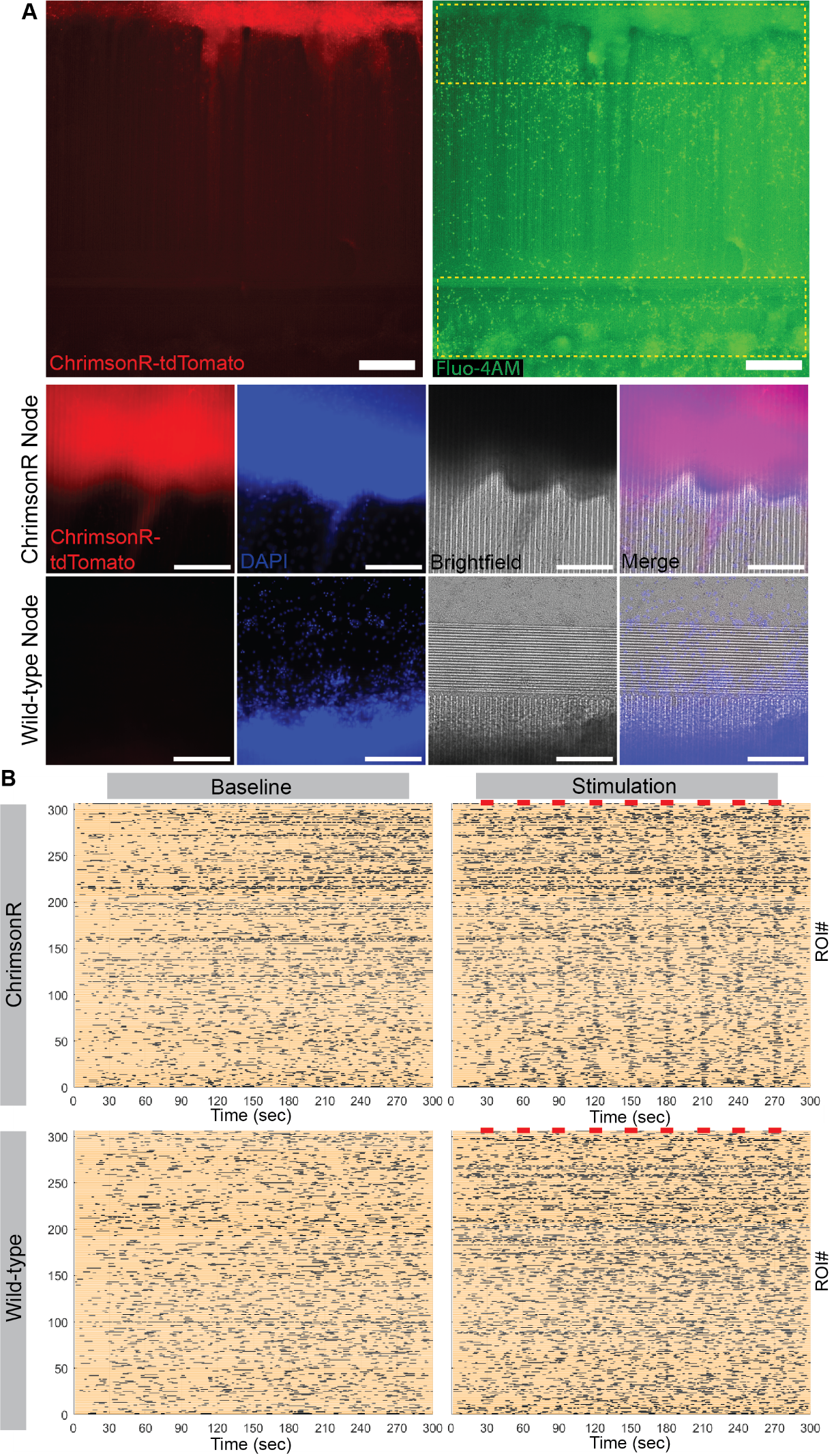


**Supplementary Figure 11**

**Optogenetic integration for neuronal activity modulation** A) Representative overview of the circuit showing ChrimsonR-expressing neurons (red, tdTomato-tagged) connected to wild-type neurons. Left: ChrimsonR-tdTomato fluorescence; right: calcium signals visualized with Fluo-4 AM (green). Rectangles indicate regions where ROIs were selected for calcium spike analysis in (B). Scale bar: 500 µm. Below: higher-magnification images of each node showing ChrimsonR-tdTomato (red), DAPI-stained nuclei (blue), and brightfield. Scale bar: 200 µm. B) Calcium traces from ROIs in both the ChrimsonR node and the wild-type node. Red dashes indicate time points when optogenetic stimulation was applied.
